# Remote ischemic conditioning protects against testicular ischemia∕reperfusion injury in rats ^1^

**DOI:** 10.1590/s0102-865020200020000003

**Published:** 2020-04-27

**Authors:** Ananda Vitória Barros Suzuki Damasceno, Charles Alberto Villacorta de Barros, Sandro Percario, Rubens Fernando Gonçalves Ribeiro, Andrew Moraes Monteiro, Eduardo Henrique Herbster Gouveia, Higor Yuri Bezerra Henriques

**Affiliations:** I Graduate student, School of Medicine, Universidade do Estado do Pará (UEPA), Belem-PA, Brazil. Care of animals, technical procedures, interpretation of data, manuscript preparation.; II PhD, Full Professor, Head, Division Surgical Abilities, UEPA, Belem-PA, Brazil. Conception, design, and scientific content of study, critical revision.; III PhD, DSc, Associate Professor, Oxidative Stress Research Lab Coordinator, UEPA, Belem-PA, Brazil. Conception and design of the study, statistical analysis, English version, critical revision.; IV Master, Postgraduate Program in Surgery and Experimental Research, UEPA, Belem-PA, Brazil. Statistical analysis, manuscript preparation, English version.; V Graduate student, UEPA, Belem-PA, Brazil. Care of the animals, technical procedures, interpretation of data, manuscript preparation.; VI MD, Graduated from Centro Universitário do Estado do Pará. Brazil. Care of animals, technical procedures, interpretation of data, manuscript preparation.

**Keywords:** Acetylcysteine, Ischemia, Reperfusion, Spermatic Cord Torsion, Testis, Rats

## Abstract

**Purpose:**

To evaluate the effect of remote ischemic conditioning associated to N-acetylcysteine (NAC) on testicular ischemia∕reperfusion (I∕R) injury in rats.

**Methods:**

Twenty-five adult male Wistar rats were randomly distributed into five experimental groups (n=5), as follows: Sham, I∕R, Perconditioning (PER), NAC and PER+NAC. Two-hour ischemia was induced by rotating the left testis 720° to clockwise direction, followed by 4 hours of reperfusion. Perconditioning was performed by three I/R cycles of 10 min each on the left limb, 30 min before reperfusion. N-acetylcysteine (150 mg∕kg) was administered 30 min before reperfusion.

**Results:**

Statistical differences were observed in MDA levels between I/R group with all groups (p<0.01), in addition there was statistical difference between PER and Sham, and PER+ NAC groups (p<0.05) in plasma.

**Conclusions:**

The protective effect of perconditioning isolated in the reduction of lipid peroxidation related to oxidative stress was demonstrated. However, when Perconditioning was associated with NAC, there was no protective effect against testicular injury of ischemia and reperfusion.

## Introduction

Torsion of the spermatic cord is a common urologic emergency among infants and adolescents. It requires early diagnosis and surgical intervention to prevent subfertility and infertility^1^ . Testicular injury resulting from spermatic cord torsion/detorsion resembles the ischemia/reperfusion phenomenon (I/R)^2^ . Moreover, it has been demonstrated that spermatic cord torsion in rats causes permanent aspermatogenesis^3^ .

This loss of spermatogenesis has shown to be due to germ cell-specific apoptosis. I/R of the testis stimulates an intra-cellular signaling cascade in the testicular endothelial cells that results in neutrophil recruitment, increase of intra-testicular reactive oxygen species (ROS), and eventual germ cell-specific apoptosis^4^ . Although with not conclusive results, several anti-inflammatory drugs^5^ , antioxidants^6^ and ischemic conditioning have been used to prevent such I/R injury in testis^7^ .

Among these conditionings, ischemic perconditioning has been highlighted as the most promising strategy to improve tolerance to I/R injury. It consists of multiple short periods of ischemia followed by the same periods of reperfusion, applied during prolonged ischemia^8^ . This method was found to be effective in the treatment of myocardial^9^ , kidney^10^ , and cerebral ischemia^11^ , through temporary and short-term enhancement of cellular antioxidant defenses to avoid the deleterious consequences of future I∕R injury^8^ .

Moreover, several studies have shown that treatment of antioxidants and free radical scavengers prevents testicular injury and male infertility^6 , 7^ . Among them, N-acetylcysteine (NAC) has been proved as a powerful antioxidant that acts as a free-radical scavenging agent and precursor of glutathione synthesis. Its powerful antioxidant features led NAC to be tested on many studies of I/R injury^12^ .

This study aimed to compare the effects of perconditioning combined to NAC on experimental testicular I/R injury through serum oxidative stress analysis.

## Methods

All experimental procedures were conducted according to international ethics guidelines and were previously locally approved by the Ethics Committee of the State University of Pará (protocol 17∕2015).

Twenty-five adult male rats aged 90 days (300-350g), were obtained from the Evandro Chagas Institute (IEC∕PARÁ). Animals were maintained with free access to regular food and water, at 22+ 1°C, under a 12h light∕dark cycle. The rats were weighed and anesthetized using an intraperitoneal injection of ketamine hydrochloride 10% and xylazine hydrochloride 2% (70 mg/kg and 10 mg/kg, respectively). During the surgical procedures, additional doses were administered if necessary^13^ .

### Experimental protocol

Five groups comprised this study:

- Sham group (SG): animals were subjected to all operative procedures, except testicular torsion;
- I∕R group: rats were submitted to 2-hours of ischemia followed by 4 hours of reperfusion^14^ ;
- Perconditioning group (PER): perconditioning was performed by three cycles of 10 min of I/R with tourniquet: 5 min under ischemia and 5 min of reperfusion on left hind limb^10^ , 30 min before reperfusion as in I/R group;
- N-acetylcysteine group (NAC): animals received an intraperitoneal injection of NAC (150 mg∕kg)^15^ , 30 min before reperfusion as in I/R group;
- Perconditioning+NAC: perconditioning was performed as in PER group and NAC administered 30 minutes before reperfusion.

### Surgical procedure

**Figure 1:**
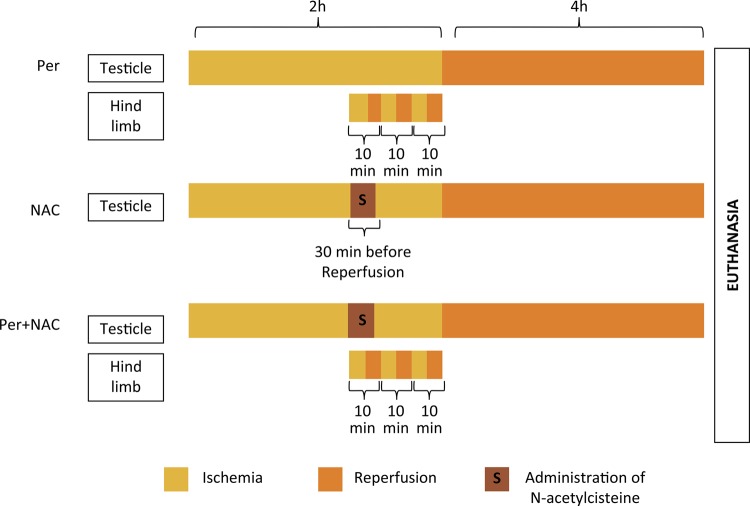
Schematic diagram of the interventions in PER (Perconditioning), NAC (administration of N-acetylcisteine) and PER+NAC (association of techniques) groups.

The surgical procedures were performed under sterile conditions through standard ilioinguinal incisions. Left testes were exposed and torsion was performed by twisting the testicular cord 720° clockwise. After 2 hours, the left testicular cord was restored to its anatomical position with the testes replaced back to their normal position. Four hours after reperfusion, rats underwent euthanasia by anesthetic overdose. PER, NAC and PER+NAC groups are better explained in Figure 1 .

### Biochemical parameters

Blood was collected by cardiac puncture just before euthanasia and serum was obtained after suitable centrifugation of blood samples and stored at -20°C until analysis.

### Thiobarbituric acid reactive substances (TBARS)

TBARS is a method that evaluates lipid peroxidation and was used as an indicator of oxidative stress. This technique is based on the reaction of malondialdehyde (MDA), among other substances, with Thiobarbituric acid (TBA; Sigma-Aldrich^®^ T5500), in low pH and high temperature, yielding MDA-TBA complex of pink colour, and absorbance peak at 535 nm. The technical procedure was performed according to the protocol proposed by Khon and Liversedge^16^ , adapted by Percario *et al.* ^17^ . In brief, initial TBA solution (10 nM) was prepared in phosphate monobasic potassium (KH_2_PO_4_ 75 mM; Synth; cat. 35.210) adjusted to pH 2.5 with acetic acid. Two-hundred and fifty μL of sample was added to 500 μL of TBA solution, mixed and placed in a water bath (95°C x 60 min); after cooling at room temperature, 2.0 ml of 1-butanol was added, vortex mixed and subsequently centrifuged (175×g×15 min); 1.0 ml of the supernatant was collected and read at 535 nm (Femto^®^, São Paulo, Brazil; 800 XI). 1,1,3,3, tetraethoxypropane (Sigma-Aldrich^®^; T9889) was used for the implementation of the standard curve.

### Trolox Equivalent Antioxidant Capacity (TEAC)

TEAC evaluates the total anti-oxidative capacity of the serum. This technique is based on the inhibition of the absorbance of the free-radical ABTS^*+^ (2,2’-Azino-bis 3-Ethylbenzthiazoline-6-Sulfonic Acid) by anti-oxidants, and on the relation of this radical cation with the anti-oxidant scavenger Trolox, which is the synthetic analogue of Vitamin E. The method modified by Re *et al* .^18^ , in brief: Trolox (Aldrich Chemical Co® 23,881-3, USA), (6-hydroxy-2,5,7,8-tetramethylchromane-2-carboxylic acid), was used as anti-oxidant. ABTS (Sigma-Aldrich®, A1888, USA) -) and potassium persulfate K_2_S_2_O_8_ (Sigma-Aldrich^®^, P5592, USA) were used to prepare a solution containing the radical cation ABTS^•+^. A saline solution (phosphate buffer PBS) prepared by diluting 1.48g Na_2_HPO_4_ (Sodium dibase phosphate), 0.43g NaH_2_PO_4_ (Sodium monobase phosphate) and 7g NaCl (potassium chloride) in 1 L of distilled water, adjusting for a pH of 7.4 was used. A calibration curve for the Trolox equivalent antioxidant capacity was built by plotting different concentrations of Trolox (mM) versus its total equivalent antioxidant capacity. The final results will be expressed in micromoles per liter (mM/l) corresponding to trolox concentration with anti-oxidant capacity equivalent to that of the studied sample, a standard of measurement called TEAC (trolox equivalent antioxidant capacity).

### Statistical analysis

All data were statistically analyzed using BioEstat^®^ 5.4 software^19^ . Data were expressed as mean + standard deviation. First, descriptive statistics were performed, showing arithmetic mean and standard deviation values. So, the normality test of Shapiro-Wilk was applied, confirming parametrical distribution of data. Finally, the Analysis of variance (ANOVA) was applied. ANOVA test was used for statistical analysis of data among all groups and *post hoc* Tukey test was applied to both variables. A value of p < 0.05 was considered statistically significant.

## Results

**Figure 2:**
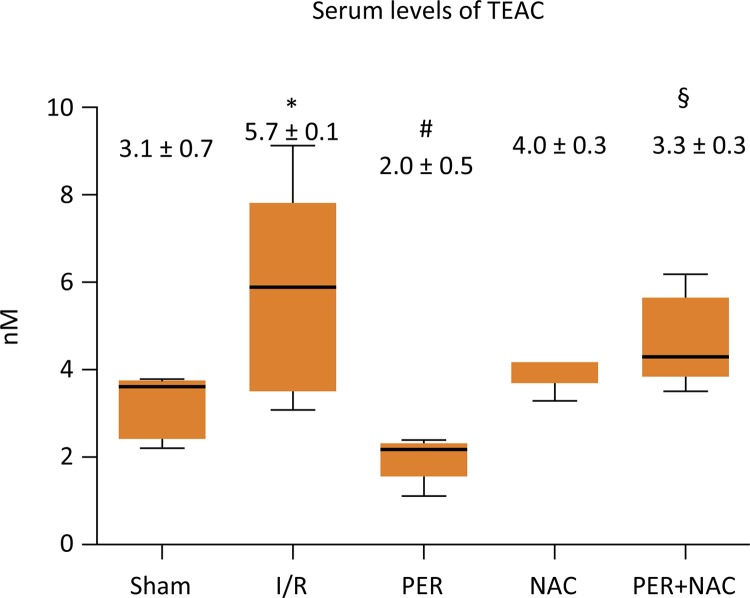
- Serum levels of MDA between groups*p<0.05 I/R *vs.* Sham group; # p<0.05 Per *vs.* I/R group; § p<0.05 Per + NAC *vs.* Per group ANOVA test (Tukey). Source: Research protocol. (MDA: Malondialdehyde).

**Figure 3:**
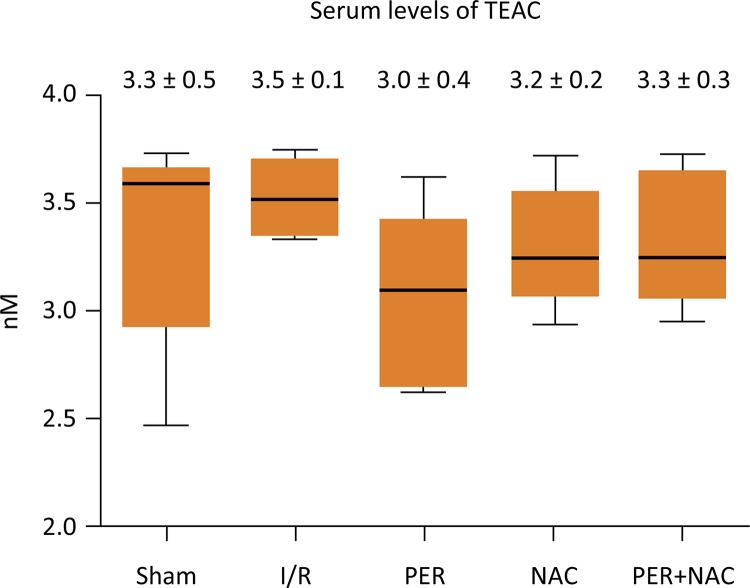
Serum levels of TEAC between groups. No significant difference (p=0.2239). ANOVA test (Tukey). Source: Research protocol. (TEAC: Trolox Equivalent Antioxidant Capacity).

Compared to Sham group, I/R showed a statistically significant difference in MDA between I/R and Sham (p=0.05); I/R and Per (p=0.01); Per and Per+NAC (p=0.05), However, There was not statistical difference between groups at serum antioxidant capacity (p=0.2239) ( Figs. 2 and 3 ).

## Discussion

On the present study, I/R group showed TBARS serum levels significantly higher than those of Sham group, demonstrating the experimental model was adequate in induction of testicular ischemic injury, promoting the oxidative pattern expected.

A parasympathetic neural response was identified as one of the effector mechanisms of ischemic conditioning techniques, reducing the lesion of non-perfusion by mechanism of vasospasm and contributing to the reestablishment of blood flow in the microcirculation of the organ submitted to I/R injury, which at the testicular level may be associated with an improvement of its physiology against oxidative damage^20 , 21^ .

It is suggested that this mechanism is the activation of a neurohumoral pathway against the reperfusion injury of organs submitted to ischemia^22^ . The addition of remote ischemic conditioning may induce the release of humoral factors, such as adenosine^23^ , bradykinin^24^ and opioids^25^ which, under local innervation, would trigger activation of neural pathways to ensure testicular protection^15^ . A recent research by Oliveira *et al* .^26^ showed tramadol (an opioid substance) had a protective effect against renal I/R oxidative injury.

A study by Guimarães *et al* .^27^ involving testicular ischemia in rats, also showed no statistical difference between the groups regarding plasma antioxidant capacity, suggesting that the torsion of the spermatic cord does not affect the systemic antioxidant system, at least under the conditions of the present research. In addition, Costa *et al* .^8^ observed in a previous study that the antioxidant capacity presented an increase of this capacity at 10 minutes, not being observed a significant increase in the time of 60 minutes, which would explain such result.

Furthermore, high mortality was observed in the animals in which NAC was used. Five animals died in the NAC group and in previous studies it has been observed that such substance promotes a decrease in prothrombin time, anaphylactic reactions and deaths^21^ . Besides that, Oliveira *et al* .^22^ demonstrated that, for the myocardium, NAC actually significantly inhibits the protective effects of ischemic conditions and could explain the difference found in TBARS levels between the conditioning group and those who were treated with NAC.

Further studies are necessary to evaluate the effects of perconditioning on reducing I/R injury, exploring neuro-humoral pathways.

## Conclusion

The perconditioning method was successful in reducing lipid peroxidation; however, NAC alone or combined to ischemic perconditioning was not effective to reduce the effects of oxidative stress.

## Footnotes

1 Research performed at Laboratory of Experimental Surgery, Universidade Estadual do Pará (UEPA), Belem-PA, Brazil.
